# Nonalcoholic Fatty Liver Disease and Hypothyroidism: What You Need to Know

**DOI:** 10.7759/cureus.28052

**Published:** 2022-08-16

**Authors:** Viktoriya Bikeyeva, Ahmed Abdullah, Aleksandra Radivojevic, Anas A Abu Jad, Anvesh Ravanavena, Chetna Ravindra, Emmanuelar O Igweonu-Nwakile, Safina Ali, Salomi Paul, Shreyas Yakkali, Sneha Teresa Selvin, Sonu Thomas, Pousette Hamid

**Affiliations:** 1 Internal Medicine, California Institute of Behavioral Neurosciences & Psychology, Fairfield, USA; 2 Behavioral Neurosciences and Psychology, California Institute of Behavioral Neurosciences & Psychology, Fairfield, USA; 3 General Surgery, California Institute of Behavioral Neurosciences & Psychology, Fairfield, USA; 4 Medicine, California Institute of Behavioral Neurosciences & Psychology, Fairfield, USA; 5 Neurology, California Institute of Behavioral Neurosciences & Psychology, Fairfield, USA

**Keywords:** thyroid-liver axis, thyroid hormones, nonalcoholic fatty liver disease (nafld), hypothyroidism, hypothyroidism-induced nafld

## Abstract

Non-alcoholic fatty liver disease (NAFLD) represents one of the leading causes of chronic liver disease globally, perhaps because of the drastic increase in prevalence around the world during the last 20 years and continues growing. The disease starts from simple steatosis (NAFL) that can progress to non-alcoholic steatohepatitis (NASH) and, in some patients, progress to cirrhosis and hepatocellular carcinoma (HCC). The pathogenesis and pathophysiology of NAFLD are complex and involve different factors (genetic, metabolic, endocrinopathies, and others). One of the concerns that appeared in recent years is hypothyroidism-induced NAFLD. The pathogenesis is compound and not well understood, and an association between hypothyroidism and NAFLD remains controversial because of insufficient studies that can confirm it. More research is needed to determine the association between hypothyroidism and NAFLD and the underlying mechanisms. In this review, we will discuss a more in-depth analysis of the physiology of thyroid hormones (TH) as well as the pathophysiology of hypothyroidism-induced NAFLD and, based on the recent meta-analyses, the association of thyroid hormones and NAFLD.

## Introduction and background

Non-alcoholic fatty liver disease (NAFLD) is a form of chronic liver disease increasingly arising among children and adults. It ranges from simple steatosis and non-alcoholic steatohepatitis (NASH) to advanced fibrosis and cirrhosis with chronic liver failure. Non-alcoholic fatty liver disease is a condition marked by the deposition of lipids in the liver cells in patients who consume very little or no alcohol. The histological picture is similar to alcohol-induced liver injury, yet it occurs in people who do not drink or consume very little. Liver enzymes such as alanine aminotransferase (ALT), aspartate aminotransferase (AST), gamma-glutamyl transferase (GGT), and other markers of liver injury are increased in NAFLD. However, those levels are normal in a large percentage of patients with NAFLD [1,2].

According to a meta-analysis that included more than eight and a half million people from 22 countries, the worldwide prevalence of fatty liver is 24%, whereas, in the United States, the prevalence of NAFLD is also 24% [3]. Compared to a worldwide population, patients with NAFLD and non-alcoholic steatohepatitis have an increased death rate due to liver-related diseases. These patients are strongly associated with extrahepatic diseases such as endocrinopathies like diabetes mellitus, metabolic syndrome, thyroid dysfunction, insulin resistance, cardiovascular diseases, and chronic kidney diseases [3].

Subclinical hypothyroidism is characterized by increased plasma thyroid-stimulating hormone (TSH) and plasma thyroid hormone levels within the reference range and without obvious clinical symptoms. Overt hypothyroidism has obvious clinical symptoms and low free Thyroxine (fT4) [4]. Thyroid hormones are crucial in multiple physiological processes like homeostasis, mineral, lipid, carbohydrates, and protein metabolisms. Lipid metabolism has been reported, such as increased metabolic rate, weight loss, lipolysis, and lowering serum cholesterol levels. The physiological process of thyroid hormones (TH) affects almost every organ, and the liver is one of the most critical targets of TH [5]. Low thyroid hormone function may cause hypercholesterolemia which plays a fundamental role in the pathophysiology of hypothyroidism-induced NAFLD [6].

In recent years, more attention has been brought to hypothyroidism-induced NAFLD. The pathogenesis of non-alcoholic fatty liver disease and thyroid hormones may have close correlations, as research in the last ten years showed that disruptions of cellular TH signaling, trigger chronic hepatic disease, including non-alcoholic fatty liver disease, alcoholic fatty liver disease, and hepatocellular carcinoma. This disruption results in decreased hepatic lipid utilization and secondary lipid accumulation [3,5,7].

The present review will discuss associations between hypothyroidism and NAFLD and the importance of thyroxine (T4), triiodothyronine (T3), and thyroid-stimulating hormone (TSH) in its pathophysiology. The association between NAFLD and hypothyroidism is still controversial. At present, there are multiple studies showing an association. Therefore, it is necessary to conduct more studies in order to answer this question [8,9]. 

## Review

Since discovering alcohol-related liver disease in 1845, fatty liver disease (FLD) has been primarily linked to excessive alcohol consumption. Non-alcoholic fatty liver disease was first described in 1981, and it includes a wide variety of hepatic conditions that include simple steatosis to steatohepatitis, advanced fibrosis, and cirrhosis. Non-alcoholic steatohepatitis and NAFLD have a similar presentation on histology, but in the case of NAFLD, there is different pathophysiology [10].

The prevalence of NAFLD among the population drastically increased in the last twenty years. NAFLD manifests itself in various ways in people worldwide, affecting both the female and male sex. The global prevalence of NAFLD is 25 percent, which is approximately as high as one billion and growing. One of the most common causes of chronic liver disease in the United States is NAFLD. It affects 80 to 100 million people, and approximately 25% of the cases progress to NASH. NAFLD includes a wide range of histopathological conditions such as non-alcoholic fatty liver (NAFL), non-alcoholic steatohepatitis (NASH), fibrosis, NASH cirrhosis, and NASH-related hepatocellular carcinoma (HCC). NAFLD is a diagnosis of exclusion. The number of NASH patients with cirrhosis is rising, resulting in an increase in liver transplantation for end-stage cirrhosis [11-13].

NAFLD is characterized by the accumulation of more than five percent of hepatic fat in the absence of any secondary causes. NAFLD can manifest in different ways, ranging from a simple accumulation of fat that is a metabolic condition with no symptoms and no inflammation of the liver (non-alcoholic fatty liver) to symptomatic non-alcoholic steatohepatitis with inflammation of the liver. It has the capacity to progress into severe fibrosis, end-stage liver disease, and HCC depending on NAFLD subtypes. While 10 to 20% of NAFLD patients develop NASH, only zero to four percent develop cirrhosis within 10 to 20 years. The rising prevalence of NAFLD with advanced fibrosis is concerning because individuals with advanced fibrosis tend to have an increased death rate of liver-related and non-liver-related diseases than the general population [11,14-17]. 

The spectrum of NAFLD 

NAFLD is divided into four stages: 1. Simple steatosis (NAFL) - accumulation of fat in the liver without inflammation or damage to the liver cells; 2. NASH - accumulation of fat in the liver with the presence of inflammation and liver cell damage - hepatitis; 3. Fibrosis - scarring of the liver tissue (excess of fibrous tissue) in the inflamed liver. Fibrosis is categorized into stages - mild, moderate, and advanced, or into four stages (0-4) based on the progression of scaring; 4. Liver cirrhosis - permanently damage to the liver with nodules of damaged liver cells surrounded by scar tissue; 5. Progression to HCC [18].

Etiology

To diagnose NAFLD, we must exclude other causes such as alcohol abuse (which is defined as consumption of >20 g/day for women or >30 g/day for men), drug abuse, Hepatitis C and Hepatitis B, Wilson's disease, hemochromatosis, celiac disease, and autoimmune liver disease, because NAFLD is a diagnosis of exclusion [19,20]. 

Insulin resistance and obesity are the two most important risk factors for NAFLD. However, NAFLD is associated with other extrahepatic manifestations such as obstructive sleep apnea, hypertension, gut microbiota alterations, dyslipidemia, sedentary lifestyle, overconsumption of carbohydrates (leading to de novo lipogenesis), and genetic predisposition. The prevalence of NAFLD in obese patients is 51%, patients with dyslipidemia are 69%, Type 2 diabetes 22,5%, and patients with hypertension are 39,3%, respectively. Nonetheless, there are other endocrine diseases that are associated with NAFLD: hypopituitarism, hypogonadism, polycystic ovarian syndrome, and hypothyroidism [11,14,19].

Thyroid hormones production and the role of thyroid hormones in the liver 

Thyroxine (T4 or 3,3′,5,5′-tetraiodo-l-thyronine) and triiodothyronine (T3 or 3,5,3′-triiodo-l-thyronine) are thyroid hormones. The thyroid gland produces predominantly T4, but deiodination of T4 in peripheral tissues creates the majority of systemic T3, which is the most potent thyroid hormone. The hypothalamic-pituitary axis controls the secretion of TH from the thyroid gland. Thyrotropin-releasing hormone (TRH) is a hormone released by the hypothalamus that acts on the pituitary gland by binding to G protein-coupled TRH receptors on the thyrotrope, causing an increase in intracellular cyclic adenosine monophosphate (cAMP) and the production of thyrotropin. TSH stimulates the generation and release of TH by binding to a G protein-coupled TSH receptor on the thyroid follicular cell [21,22]. 

The thyroid gland regulates many biological activities in the liver, adipose tissue, central nervous, cardiovascular, and musculoskeletal systems by producing and releasing thyroid hormones such as thyroxine and triiodothyronine into the blood. One of the main physiological functions of TH is to maintain basal energy expenditure by glucose and lipid metabolism modulation. Thyroid hormones increase the production of fatty acids, modulate the sensitivity of the insulin in the hepatic tissue, and decrease hepatic gluconeogenesis, in addition to increasing lipid export and oxidation. The TH receptor (TR) regulates lipid metabolism (synthesis, mobilization, and degradation) as well as the metabolism of glucose by regulating the expression of several other nuclear receptors. HMG-CoA reductase (3-hydroxy-3methylglutarylcoenzyme A), which initiates cholesterol biosynthesis, can be stimulated by thyroid hormones. Also, triiodothyronine (T3) has the ability to bind to certain thyroid hormone-responsive elements and activate the LDL receptor gene, thereby upregulating LDL receptors. Also, thyroid hormones regulate HDL metabolism. Thyroid hormones increase the expression of the regulatory sterol element-binding protein-2, which regulates cholesterol metabolism (SREBP-2). T3 also stimulates lipoprotein lipase, which catabolizes triglyceride (TG)-rich lipoproteins, resulting in a reduction in TG levels [15,22-25].

Both T3 and T4 act via TRs, when thyroid hormones binding to the genes of thyroid receptors, which then help facilitate the transport of free fatty acids in the cells of the hepatic tissue, with the help of protein transporters like liver fatty acid-binding proteins (L-FABPs), fatty acid transporter proteins (FATPs), and fatty acid translocases (FAT). THs can increase intrahepatic lipolysis through lipophagy in hepatocytes via THR-β, resulting in decreased TG clearance and increased TG hepatic uptake [10,26]. This type of lipophagy is linked to triiodothyronine physiology. T3 plays a significant role in fatty acid transport to mitochondria and mitochondrial metabolism by altering the range of hepatic lipid-related metabolites T3. T3 stimulates lipophagy in cultured hepatic cell lines, which leads to hepatic autophagy and ketogenesis. These results suggest that T3 regulates hepatic autophagy, which is an important step in the management of NAFLD [27].

Hypothyroidism induced NAFLD 

We know that hypothyroidism is linked to hypometabolism. It is characterized by increasing weight, decreasing resting energy expenditure, and decreasing gluconeogenesis and lipolysis. Obesity, impaired lipid metabolism, and insulin resistance can be caused by THs dysfunction, which are symptoms of metabolic syndrome that are also present in NAFLD [28]. Overt hypothyroidism and subclinical hypothyroidism are both related to NAFLD [8].

In subclinical hypothyroidism, there are several factors that contribute to the progression of NAFLD, such as the physiology of TSH on the hepatocytes cell membrane, impairing hepatic triglyceride metabolism (promoting hepatic lipogenesis) through the upregulation of SREBP-1c activity provoked by stimulation of TSH receptors. Also, in the case of low thyroid hormone, diminished glucose-sensing receptors of the beta cells of the pancreas. In this manner, it reduces insulin secretion and decreases lipolysis in the adipose tissue, increasing the traffic of FFA to the hepatic tissue [19,22,29].

Patients with hypothyroidism often have atherogenic dyslipidemia. It has been established that the pathophysiology of hypothyroidism-induced hyperlipidemia is due to a reduction in cholesterol excretion and a marked rise in apoB lipoproteins as a result of insufficient catabolism and turnover brought on by a reduction in the number of low-density lipoprotein (LDL) receptors on the surface of the hepatic cells. In this manner, elevated total and LDL cholesterol levels are common in hypothyroid patients. Also, decreased clearance level of triglycerides from plasma and the buildup of intermediate density lipoproteins were noted in hypothyroidism. Hypothyroidism-induced NAFLD may develop because of the rising LDL and accumulation of triglycerides in the hepatic tissue [22,27]. Accumulation of the lipids causes oxidative stress and inflammatory response in the liver [8]. Another factor that may be involved in the thyroid-liver complex is leptin. Leptin is elevated in hypothyroid patients and is also elevated in NAFLD patients. Leptin can promote hepatic insulin resistance and play a role in hepatic fibrogenesis [7].

In the meta-analysis from 2018 that involved 26 studies and 61,548 participants, 11 studies with a total of 47,217 patients with NAFLD/NASH had significantly higher thyroid-stimulating hormones than healthy controls, this difference remains significant. With the progression of NAFLD, the level of TSH increased as well. The research found that hypothyroidism raised the probability of non-alcoholic fatty liver disease or non-alcoholic steatohepatitis. These results were disputed in subsequent evaluations based on the degree of hypothyroidism. The risk of non-alcoholic steatohepatitis was substantially correlated with subclinical hypothyroidism but not with the risk of NAFLD. On the other hand, the risk of non-alcoholic fatty liver disease is substantially correlated with overt hypothyroidism in adults but not with the risk of NASH. These results might be inconsistent due to the small number of included studies. This meta-analysis also discovered that the relationship between NAFLD and free T3 (FT3) and free T4 (FT4) may vary by the number of people that live in the area and that non-alcoholic fatty liver disease is perhaps unrelated to FT3 or FT4. These results might be evidence that TSH, rather than thyroid hormones, plays a key role in the onset and progression of NAFLD [8].

In another meta-analysis from 2021 that involved 17 articles and 14,514 participants included, elevated TSH levels maybe be a risk factor that is independently associated with NAFLD. FT4 was significantly associated with NAFLD when FT3 was not associated [30].

Treatment of hypothyroidism-induced NAFLD

There is no drug therapy for hypothyroidism-induced NAFLD that is currently approved. Steatosis can be reduced through structured lifestyle changes such as weight loss, dietary changes such as reduced drinking of alcohol, decreasing intake of food and drinks that have a high level of fructose, and increased daily activities and workouts [16,29]. 

In a case report of a patient with NASH who was diagnosed with Graves' disease (GD), the liver enzyme levels improved after the onset of GD and subsequent hyperthyroidism. They worsened after starting treatment and returning to a euthyroid state [31].

A small-molecule liver-directed thyroid hormone receptor β agonist (Resmetirom) with high liver uptake is administered orally and is under development (currently in Phase 3 of trial) for the treatment of NAFLD/NASH and familial hypercholesterolemia [4,32]. 

## Conclusions

During the last twenty years, the prevalence of NAFLD increased significantly, and understanding this disease has become crucial. Due to rising concerns about hypothyroidism-induced NAFLD and for better management of this condition, knowing the pathophysiology is critical. Thyroid hormones play a vital role in the thyroid-liver axis and lipid metabolism. Disruption of the lipid metabolism can cause lipids accumulation with a subsequent inflammatory response in the liver and cause NAFLD. There is no current approved FDA treatment for hypothyroidism-induced NAFLD, but they are under development, and the pathophysiology of this disease is essential for correct treatment. We reviewed the two biggest meta-analyses from 2018 and 2021. In both analyses, it was found that an increased level of TSH may be a risk for NAFLD. Nevertheless, FT3 and FT4 are still controversial because they showed heterogenic results. Large-designed well-scaled prospective studies need to be done to confirm current findings and better define the role of FT3 and FT4 in hypothyroidism-induced NAFLD.

## References

[1] Norbert Stefan, Hans-Ulrich Häring (2005). Nonalcoholic fatty liver disease: a new entry in the metabolic syndrome?. The metabolic syndrome at the beginning of the XXI century: a genetic and molecular approach.

[2] Anna Wieckowska, Ariel E. Feldstein (2011). Nonalcoholic fatty liver disease. Pediatric gastrointestinal and liver disease (fourth edition).

[3] Guo Z, Li M, Han B, Qi X (2018). Association of non-alcoholic fatty liver disease with thyroid function: a systematic review and meta-analysis. Dig Liver Dis.

[4] Tanase DM, Gosav EM, Neculae E, Costea CF, Ciocoiu M, Hurjui LL (2020). Hypothyroidism-induced nonalcoholic fatty liver disease (HIN): mechanisms and emerging therapeutic options. International Journal of Molecular Sciences.

[5] Kowalik MA, Columbano A, Perra A (2018). Thyroid hormones, thyromimetics and their metabolites in the treatment of liver disease. Front Endocrinol (Lausanne).

[6] Chung GE, Kim D, Kim W (2012). Non-alcoholic fatty liver disease across the spectrum of hypothyroidism. J Hepatol.

[7] Mandato C, D'Acunzo I, Vajro P (2018). Thyroid dysfunction and its role as a risk factor for non-alcoholic fatty liver disease: What's new. Dig Liver Dis.

[8] He W, An X, Li L, Shao X, Li Q, Yao Q, Zhang JA (2017). Relationship between hypothyroidism and non-alcoholic fatty liver disease: a systematic review and meta-analysis. Front Endocrinol (Lausanne).

[9] Jaruvongvanich V, Sanguankeo A, Upala S (2017). Nonalcoholic fatty liver disease is not associated with thyroid hormone levels and hypothyroidism: a systematic review and meta-analysis. Eur Thyroid J.

[10] Chi HC, Chen CY, Tsai MM, Tsai CY, Lin KH (2013). Molecular functions of thyroid hormones and their clinical significance in liver-related diseases. Biomed Res Int.

[11] Perumpail BJ, Khan MA, Yoo ER, Cholankeril G, Kim D, Ahmed A (2017). Clinical epidemiology and disease burden of nonalcoholic fatty liver disease. World J Gastroenterol.

[12] Younossi ZM, Koenig AB, Abdelatif D, Fazel Y, Henry L, Wymer M (2016). Global epidemiology of nonalcoholic fatty liver disease- meta-analytic assessment of prevalence, incidence, and outcomes. Hepatology.

[13] Zhu B, Chan SL, Li J, Li K, Wu H, Cui K, Chen H (2021). Non-alcoholic steatohepatitis pathogenesis, diagnosis, and treatment. Front Cardiovasc Med.

[14] Juanola O, Martínez-López S, Francés R, Gómez-Hurtado I (2021). Non-alcoholic fatty liver disease: metabolic, genetic, epigenetic and environmental risk factors. Int J Environ Res Public Health.

[15] Lai S, Li J, Wang Z, Wang W, Guan H (2021). Sensitivity to thyroid hormone indices are closely associated with NAFLD. Front Endocrinol (Lausanne).

[16] Sanyal D, Mukherjee P, Raychaudhuri M, Ghosh S, Mukherjee S, Chowdhury S (2015). Profile of liver enzymes in non-alcoholic fatty liver disease in patients with impaired glucose tolerance and newly detected untreated type 2 diabetes. Indian J Endocrinol Metab.

[17] Cariou B, Byrne CD, Loomba R, Sanyal AJ (2021). Nonalcoholic fatty liver disease as a metabolic disease in humans: a literature review. Diabetes Obes Metab.

[18] Wang J, He W, Tsai PJ, Chen PH, Ye M, Guo J, Su Z (2020). Mutual interaction between endoplasmic reticulum and mitochondria in nonalcoholic fatty liver disease. Lipids Health Dis.

[19] Gariani K, Jornayvaz FR (2021). Pathophysiology of NASH in endocrine diseases. Endocr Connect.

[20] Marchisello S, Di Pino A, Scicali R, Urbano F, Piro S, Purrello F, Rabuazzo AM (2019). Pathophysiological, molecular and therapeutic issues of nonalcoholic fatty liver disease: an overview. Int J Mol Sci.

[21] Mullur R, Liu YY, Brent GA (2014). Thyroid hormone regulation of metabolism. Physiol Rev.

[22] Lonardo A, Ballestri S, Mantovani A, Nascimbeni F, Lugari S, Targher G (2019). Pathogenesis of hypothyroidism-induced NAFLD: evidence for a distinct disease entity?. Dig Liver Dis.

[23] Damiano F, Rochira A, Gnoni A, Siculella L (2017). Action of thyroid hormones, T3 and T2, on hepatic fatty acids: differences in metabolic effects and molecular mechanisms. Int J Mol Sci.

[24] Jung KY, Ahn HY, Han SK, Park YJ, Cho BY, Moon MK (2017). Association between thyroid function and lipid profiles, apolipoproteins, and high-density lipoprotein function. J Clin Lipidol.

[25] Shin DJ, Osborne TF (2003). Thyroid hormone regulation and cholesterol metabolism are connected through sterol regulatory element-binding protein-2 (SREBP-2). J Biol Chem.

[26] Sinha RA, Singh BK, Yen PM (2018). Direct effects of thyroid hormones on hepatic lipid metabolism. Nat Rev Endocrinol.

[27] Huang YY, Gusdon AM, Qu S (2013). Cross-talk between the thyroid and liver: a new target for nonalcoholic fatty liver disease treatment. World J Gastroenterol.

[28] Notariza KR, Wisnu W (2019). The risk of developing non-alcoholic fatty liver disease in adult patients with subclinical hypothyroidism compared to euthyroid: an evidence-based case report. Acta Med Indones.

[29] Liebe R, Esposito I, Bock HH (2021). Diagnosis and management of secondary causes of steatohepatitis. J Hepatol.

[30] Zeng X, Li B, Zou Y (2021). The relationship between non-alcoholic fatty liver disease and hypothyroidism: a systematic review and meta-analysis. Medicine (Baltimore).

[31] Von-Hafe M, Borges-Canha M, Vale C, Leite AR, Sérgio Neves J, Carvalho D, Leite-Moreira A (2022). Nonalcoholic fatty liver disease and endocrine axes-a scoping review. Metabolites.

[32] Kim D, Kim W, Joo SK, Bae JM, Kim JH, Ahmed A (2018). Subclinical hypothyroidism and low-normal thyroid function are associated with nonalcoholic steatohepatitis and fibrosis. Clin Gastroenterol Hepatol.

